# Opsoclonus-myoclonus-ataxia Syndrome in an ART-naïve Patient With CSF/Plasma HIV-1 RNA Discordance

**DOI:** 10.1093/ofid/ofaf517

**Published:** 2025-08-21

**Authors:** Hiroo Matsuo, Kairi Yonekura, Satoshi Kutsuna

**Affiliations:** Department of Infection Control and Prevention, Graduate School of Medicine, University of Osaka, Osaka, Japan; Infection Control Team, Human Resource Development Division, Center for Infectious Disease Education and Research, The University of Osaka, Osaka, Japan; Department of General Internal Medicine, Hyogo Prefectural Amagasaki General Medical Center, Hyogo, Japan; Department of Infection Control and Prevention, Graduate School of Medicine, University of Osaka, Osaka, Japan; Infection Control Team, Human Resource Development Division, Center for Infectious Disease Education and Research, The University of Osaka, Osaka, Japan

**Keywords:** antiretroviral therapy, cerebrospinal fluid, CSF/plasma HIV-1 RNA discordance, HIV infections, opsoclonus-myoclonus-ataxia syndrome

## Abstract

Opsoclonus-myoclonus-ataxia syndrome (OMAS) is a rare neuroimmunological disorder with diverse etiologies, including infection-associated triggers such as human immunodeficiency virus (HIV) infection. Herein, we report a case of HIV-associated OMAS in an antiretroviral therapy (ART)-naïve patient who presented with cerebrospinal fluid (CSF)/plasma HIV-1 RNA discordance. A 16-year-old female was diagnosed with HIV following the onset of OMAS. The CSF HIV-1 RNA level was higher than that in the plasma. After ART initiation, both the CSF HIV viral load and OMAS symptoms improved simultaneously. This case demonstrates that CSF/plasma HIV-1 RNA discordance can occur in ART-naïve patients with HIV-associated OMAS. Moreover, our findings suggest that, in such cases, viral suppression through ART alone may be sufficient to achieve clinical resolution without the need for adjunctive immunosuppressive therapy.

Opsoclonus-myoclonus-ataxia syndrome (OMAS), also referred to as “dancing eyes, dancing feet” syndrome, is characterized by a triad of opsoclonus, characterized by sudden, involuntary, high-frequency horizontal, vertical, and torsional eye movements; myoclonus, marked by a nonepileptic involuntary movement involving the limbs and trunk; and ataxia [1, 2]. The first adult case was reported in 1913 [3], followed by the first pediatric case in 1962 [4].

The etiologies of OMAS include paraneoplastic, infectious, drug- or metabolism-related, structural intracranial lesions, and idiopathic causes [1, 5]. Among the infectious triggers, viral infections, such as the Epstein–Barr virus (EBV), Coxsackie B3 virus, severe acute respiratory syndrome coronavirus 2, and human immunodeficiency virus (HIV), have been implicated [5–10]. HIV-associated OMAS has been reported in contexts, such as acute HIV infection, immune reconstitution inflammatory syndrome (IRIS), and opportunistic infections [11, 12]. Cerebrospinal fluid (CSF) HIV RNA escape, which is the persistence of viral replication in the central nervous system (CNS) despite a suppressed plasma viral load, is a proposed mechanism of HIV-related OMAS [13]. CSF HIV RNA escape and CSF/plasma HIV RNA discordance, both distinct concepts, do not have universally accepted definitions, and threshold criteria vary depending on treatment status, clinical context, and study objectives [14]. CSF HIV RNA escape is defined as the presence of quantifiable HIV RNA in the CSF despite suppressed plasma viral load, whereas discordance refers to any situation wherein the CSF HIV RNA level exceeds that in plasma, regardless of the antiretroviral therapy (ART) status [15, 16]. Thresholds for defining discordance have ranged from >0.5 to >1 log₁₀ copies/mL [16–18]. In ART-naïve individuals, a CSF/plasma HIV RNA ratio ≥1 has been proposed as a clinically relevant threshold, given its association with CNS inflammation, blood–brain barrier dysfunction, and increased risk of neurological coinfections [19, 20]; this definition was adopted was also adopted for the present case (CSF/plasma HIV RNA ratio >1) [21].

However, to date, no report has demonstrated an association between OMAS and pre-ART CSF/plasma HIV RNA discordance. Previous reports have described OMAS in the context of HIV infection, often associated with CSF HIV RNA escape during ART but not before treatment initiation [11, 13, 22–25].

Here, we present a rare case of OMAS in an ART-naïve patient with HIV in whom CSF/plasma discordance was confirmed before treatment initiation. This case suggests that CSF/plasma discordance may play a role in OMAS pathogenesis, even in untreated patients with HIV [26].

## THE CASE

### Informed Consent Statement

Informed consent for publication was obtained from the patient and her family member, and documented per hospital regulations.

### Patient Information

A 16-year-old Nepalese female with no significant medical history presented with acute walking difficulties following the sudden onset of generalized myoclonic jerks, dizziness, and oscillopsia on day X. The patient was admitted on day X + 6 for further evaluation.

### Physical Examination and Clinical Findings

On admission, the patient had stable vital signs and was alert and fully conscious. Neurological examination revealed slurred speech; wide, erratic, multidirectional spontaneous eye movements without an intersaccadic interval; a wide-based, severely unsteady gait requiring occasional assistance; and generalized myoclonic jerks, predominantly affecting the upper limbs. Multiple molluscum contagiosum lesions on the dorsal aspects of both hands with gradual progression over the last 5-years An unintentional 4 kg weight loss over the preceding 5 months suggested chronic HIV infection.

### Laboratory and Imaging Findings

On day X + 10, blood tests revealed 8.1 × 10⁴ copies/mL of HIV RNA and a cluster of differentiation 4 (CD4) count of 131 cells/μL. Western blot analysis demonstrated a complete band pattern, including env (gp160, gp110/120, gp41), gag (p55, p40, p24/25, and p18/17), and pol (p68/66, p52/51, and p34/31) proteins, consistent with chronic-phase HIV infection. Liver and renal functions were normal (CRP, 0.01 mg/dL; leukocyte count, 5.2 × 10³/μL; hemoglobin, 11.3 g/dL; platelet count, 239 × 10³/μL). Serum cryptococcal antigen testing was negative. Serological testing revealed positive *Toxoplasma gondii* IgG (473 IU/mL); further details regarding syphilis testing are provided below. CSF analysis on the same day showed an HIV-1 RNA level of 1.5 × 10⁵ copies/mL, exceeding the plasma level. The CSF showed pleocytosis (30 cells/μL, all mononuclear) with elevated protein (170.6 mg/dL) and normal glucose (48 mg/dL) levels; the corresponding serum glucose level was 74 mg/dL. Quantitative polymerase chain reaction (PCR) testing for common viral pathogens including cytomegalovirus (CMV), EBV, human herpes virus 6 (HHV-6), herpes simplex virus (HSV), varicella zoster virus (VZV), and HIV-1, was performed using assays validated for clinical use in Japan. Additional qualitative multiplex PCR testing was conducted using an in-house assay targeting HSV-1, HSV-2, VZV, CMV, EBV, HHV-6, HHV-7, HHV-8, BK virus, JC virus, adenovirus, parvovirus B19, and hepatitis B virus (HBV). All CSF PCR results were negative, except for HIV-1. The CSF cryptococcal antigen test result and anti-NMDA receptor antibody testing were both negative.

Serological testing for syphilis revealed a positive Treponema pallidum antibody titer of 1.5, negative rapid plasma reagin tests in both serum and CSF, and a negative CSF fluorescent treponemal antibody absorption (FTA-ABS) test. Tuberculosis (TB) screening revealed a negative T-SPOT. TB assay results and a normal CSF adenosine deaminase level (9.4 U/L). The CSF cultures were negative for both routine bacteria and mycobacteria. Serum paraneoplastic autoantibody screening was performed using a commercially available panel assay validated for clinical use in Japan. The panel included antibodies against amphiphysin, Yo, Hu, Ri, CV2, PNMA2, recoverin, SOX1, titin, Zic4, GAD65, and Tr (DNER). Only the antiamphiphysin antibody was weakly positive; all others were negative. These results strongly indicated that HIV-1 as the sole cause of CNS involvement.

**Figure 1.**
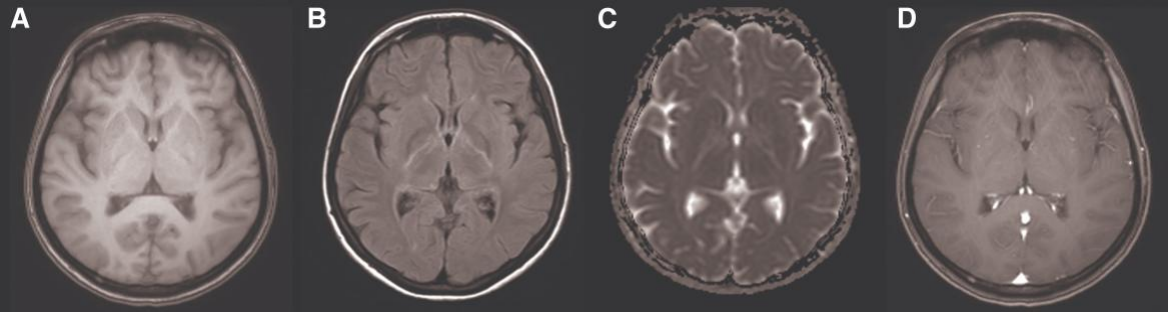
Brain MRI findings axial magnetic resonance imaging (MRI) performed on day X + 6 (the day of admission) and day X + 14 showed no space-occupying lesions, hemorrhage, or abnormal enhancement. FLAIR sequences from day X + 6 demonstrated subtle hyperintense signals in the bilateral posterior limbs of the internal capsules, corona radiata, pons, and midbrain. These findings were interpreted as possibly consistent with HIV-associated viral encephalitis. Images from left to right: (*A–C*) T1-weighted, FLAIR-T2, and ADC map (all acquired on day X + 6); (*D*) contrast-enhanced T1-weighted (T1 + Gad, acquired on day X + 14). Abbreviation: MRI, magnetic resonance imaging.

Brain magnetic resonance imaging (MRI) revealed no mass lesions, but demonstrated faint FLAIR hyperintensities in the bilateral posterior limbs of the internal capsules, corona radiata, pons, and midbrain. (Figure 1) Cervical spine MRI was unremarkable, and chest and abdominal computed tomography showed no abnormalities, including no evidence of ovarian tumors. These findings led to the diagnosis of HIV-associated OMAS.

### Therapeutic Intervention

ART with emtricitabine/tenofovir alafenamide (FTC/TAF) and dolutegravir (DTG) was initiated on day X + 14.

### Outcomes

**Figure 2.**
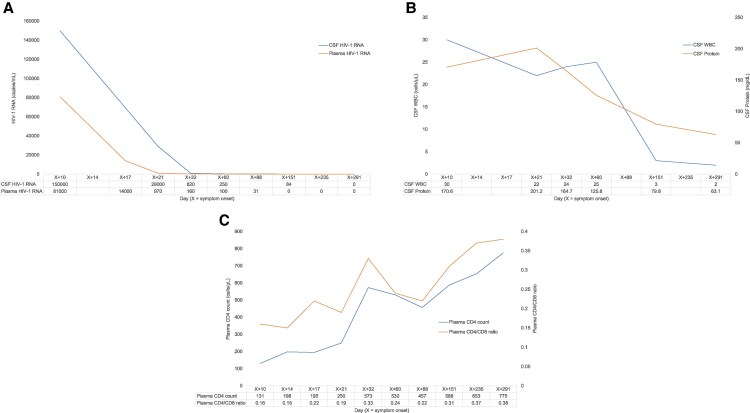
Longitudinal changes in HIV-1 RNA levels, CSF parameters, and peripheral immune markers following ART initiation. “X” denotes the day of symptom onset. All measurements were obtained from day X + 10, the day of HIV diagnosis, and followed longitudinally over the treatment course. Antiretroviral therapy (ART) was initiated on day X + 14. (*A*) HIV-1 RNA levels in plasma and cerebrospinal fluid (CSF). Plasma levels declined more rapidly than CSF, with persistently elevated CSF levels indicating delayed viral clearance in the central nervous system. (*B*) CSF white blood cell (WBC) count and protein concentration. Both parameters decreased steadily following ART initiation, with near normalization observed during follow-up. (*C*) Peripheral CD4+ T cell count and CD4/CD8 ratio. A gradual increase was seen over time, consistent with immune restoration under sustained viral suppression. Abbreviations: ART, antiretroviral therapy; CSF, cerebrospinal fluid; WBC, white blood cell; HIV, human immunodeficiency virus; CNS, central nervous system.

By day X + 19, the patient had regained independent ambulation, although the patient's gait remained slightly wide-based. By day X + 21, the patient was able to walk normally without assistance, and ocular and upper limb myoclonic jerks had markedly improved. The patient was discharged on day X + 22 with a stable neurological status and continued outpatient follow-up. On day X + 42, the myoclonic jerks were nearly resolved. Following ART initiation, both the plasma and CSF HIV-1 RNA levels decreased simultaneously with clinical improvement (Figure 2). Since then, the patient has remained asymptomatic without recurrence.

## DISCUSSION

This case highlights 2 key observations. First, OMAS in ART-naïve patients with HIV may present with CSF/plasma HIV-1 RNA discordance. Second, HIV-associated OMAS may be responsive to ART alone in the context of such discordance. These 2 observations may be mechanistically interrelated, as CSF/plasma HIV-1 RNA discordance in ART-naïve individuals likely reflects active intrathecal viral replication and the associated immune dysregulation, which may be ameliorated through ART-induced suppression of CNS viral activity.

The first point concerns the presence of CSF/plasma HIV RNA discordance at the onset of OMAS, before ART initiation. CSF/plasma discordance has been implicated in HIV-related neurocognitive disorders, such as HIV-associated dementia [27, 28], and likely reflects ongoing CNS viral replication, persistent cytokine production, and neurotoxicity [29]. Even in patients with suppressed plasma HIV viral loads undergoing ART, HIV-related neurological symptoms are reportedly associated with CSF HIV RNA escape [15]. Similar mechanisms have been proposed for HIV-related OMAS [13, 30].

In the present case, the HIV-1 RNA level in the CSF exceeded that in the plasma, consistent with CSF/plasma discordance [31]. Although the CSF CD4/CD8 ratio could not be directly assessed, previous studies have shown that elevated CSF HIV RNA levels in ART-naïve individuals are associated with a disproportionate increase in CD8+ T cells and relative reduction in CD4+ T cells, reflecting a shift toward intrathecal immune dysregulation [32]. This altered immune milieu may facilitate the emergence of HIV-associated neurological syndromes including OMAS. Our case supports the hypothesis that CSF/plasma HIV RNA discordance contributes to OMAS pathogenesis, even in the absence of ART, thereby broadening the current understanding of its underlying mechanisms.

Second, ART alone may be sufficient for clinical improvement in selected cases of HIV-associated OMAS with CSF/plasma discordance. Although the underlying mechanism of OMAS remains unclear, its responsiveness to corticosteroids, intravenous immunoglobulins, rituximab, and immunosuppressive agents in other contexts suggests an immune-mediated pathophysiology [1, 33–35]. However, in infection-associated OMAS, pathogen-specific antibodies are often absent, and postinfectious immune activation has been proposed as a possible mechanism [36].

In the context of early HIV infection, CNS dysfunction has been observed, even in the absence of high CSF HIV viral loads, suggesting immune-mediated mechanisms [37]. A reduced CD4/CD8 ratio within the CNS has also been postulated to contribute to OMAS pathogenesis in HIV [11]. If CSF/plasma discordance reflects elevated intrathecal viral activity and associated immune disturbances, viral suppression via ART may be sufficient to reverse the condition. In the present case, OMAS symptoms improved rapidly following ART initiation in parallel with a decline in CSF HIV-1 RNA levels.

The treatment strategies for HIV-associated OMAS remain variable. Spontaneous resolution has been reported in cases occurring during acute HIV infection [38] and improvement following modification or intensification of ART alone in patients with CSF HIV RNA escape [30]. In contrast, immunosuppressive therapy has been used in cases suspected of IRIS [39]. Other reports have described HIV-associated OMAS cases in which ART alone was insufficient and corticosteroids were required [24], or even cases refractory to ART, corticosteroids, intravenous immunoglobulin, and gabapentin [22]. Thus, no standardized treatment approaches are currently available.

In contrast, our patient showed a marked improvement with ART alone. This suggests that, in cases of HIV-associated OMAS with confirmed CSF/plasma HIV RNA discordance, viral suppression may be sufficient to achieve clinical resolution without the need for adjunctive immunosuppressive therapy. This case highlights the importance of including HIV infection in the differential diagnosis of OMAS, particularly in younger patients presenting with systemic or cutaneous findings suggestive of chronic immunosuppression. Moreover, it highlights the clinical relevance of evaluating CSF/plasma HIV-1 RNA discordance before ART initiation, suggesting that virological dissociation may have both diagnostic and therapeutic implications in the context of HIV-associated neurological syndromes.

This study has several limitations. First, we were unable to assess the CD4/CD8 ratio in the CSF, which would have provided a more direct insight into the intrathecal immune environment. Consequently, our interpretation was based solely on virological data and we were unable to evaluate the nature of the CNS immune response. Second, it remains uncertain whether the resolution of OMAS in this case is directly attributable to ART. To our knowledge, there have been no prior reports of OMAS with CSF/plasma HIV RNA discordance in ART-naïve patients, and the natural course of these cases remains unknown. In the present case, no clinical improvement was observed before ART initiation. However, after starting ART, OMAS symptoms improved in parallel with a gradual decline in CSF HIV-1 RNA levels. Notably, viral load became undetectable at a later time point in CSF compared with plasma, further suggesting a potential causal relationship between intrathecal viral suppression and symptom resolution. Additional studies are required to confirm whether ART alone is consistently effective in such settings. Third, the timing of the HIV infection remains unknown. Although based solely on family interviews, vertical transmission was considered unlikely because both of the patient's parents were reportedly HIV-negative. The patient had never undergone HIV testing before this episode, and this was the first documented HIV diagnosis. However, a 4-kg weight loss over the preceding months and a several-year history of progressively increasing molluscum contagiosum lesions suggested that the infection was likely not recent. Further studies incorporating immunophenotyping and longitudinal clinical history are needed to clarify these aspects.

In summary, we report a rare case of OMAS as an initial manifestation of HIV infection in an ART-naïve patient with confirmed CSF/plasma HIV RNA discordance. The patient responded rapidly and completely to ART alone, supporting the hypothesis that CSF/plasma HIV RNA discordance contributes to the pathogenesis of HIV-associated OMAS and treatment with ART alone may be sufficient in selected cases.
